# Redefining psychopathology in the context of digital overload: emerging disorders in the age of information saturation

**DOI:** 10.3389/fdgth.2025.1693287

**Published:** 2025-11-12

**Authors:** Shirin Abdallah Alimour, Mohammad Alrabeei

**Affiliations:** 1 College of Education, Humanities & Social Sciences, Al Ain University, Al Ain, United Arab Emirates; 2 College of Humanities, City University, Ajman, United Arab Emirates

**Keywords:** digital overload, cognitive fragmentation, hyperconnectivity, digital-era psychopathology, personalized digital detox, mental health diagnostics

## Abstract

The accelerating integration of digital technologies with human experience has precipitated profound cognitive, emotional, and behavioral transformations, giving rise to emergent psychopathologies that remain insufficiently addressed by traditional diagnostic taxonomies. This study introduces a novel reconceptualization of mental health in the digital era, delineating four original diagnostic categories: Cognitive Fragmentation and Digital Overload Disorders, Social Media and Immersive Technology-Induced Disorders, Technology Integration Disorders, and Symptom-Driven Disorders Requiring Diagnostic Adaptation. To explore the clinical salience of these emerging constructs, a mixed-methods pilot investigation was conducted involving a cross-sectional survey of 75 licensed mental health professionals and a retrospective analysis of 225 anonymized patient records. Findings revealed substantial clinical recognition of digital-era syndromes, with Continuous Partial Attention Disorder (CPAD) and Digital Anxiety Disorder (DAD) endorsed by 85.3% (*n* = 64/75) and 82.7% (*n* = 62/75) of clinicians, respectively. Symptom severity was predominantly rated as moderate. Inferential analyses revealed a statistically significant association between years of clinical experience and recognition of AI-related psychopathologies, including AI Identity Diffusion Disorder [*χ*^2^ (1, *N* = 75) = 5.33, *p* = .021]. Chart review corroborated these findings, with 76% (*n* = 171/225) of cases documenting symptoms consistent with digital-era psychopathologies, and CPAD alone noted in 36.4% of records. These results underscore the growing clinical relevance of technology-induced mental health disorders and highlight the urgent need to evolve current diagnostic frameworks. The paper calls for the development of standardized, digitally responsive assessment tools and the design of innovative, context-sensitive therapeutic modalities. Future research should prioritize longitudinal investigations, digital phenotyping, and psychometric validation to enhance diagnostic precision and treatment effectiveness in an increasingly digitized world.

## 1 Introduction

The widespread integration of digital technologies has profoundly reshaped human life—transforming communication, access to information, and social interaction. While these innovations offer significant benefits, they have also introduced unprecedented psychological challenges. One of the most pervasive is digital overload, characterized by the relentless influx of digital stimuli and fragmented attention demands across multiple platforms. This phenomenon has been associated with cognitive strain, attentional instability, memory disruption, and affective dysregulation (1–3). Recent global data indicate that smartphone penetration has exceeded 85% worldwide, with adults spending an average of 7–9 h daily engaged with digital devices (4), underscoring the widespread exposure to these digital environments. This growing exposure has profound implications not only for cognitive functioning but also for clinical practice, public health policy, and mental health prevention strategies.

Although traditional diagnostic systems such as the DSM-5 and ICD-11 were developed to classify disorders grounded in neurochemical imbalances, cognitive distortions, and environmental stressors, they have not adequately evolved to address the psychological complexities of sustained digital immersion (5, 6). In particular, these systems lack validated criteria to identify or classify psychological conditions driven by chronic digital engagement and hyperconnectivity. This gap is increasingly evident as technology mediates not only behavior but also perception, identity, and interpersonal dynamics. Current nosologies inadequately classify emergent digital-related symptom profiles, as they lack constructs capturing psychopathologies driven by hyperconnectivity, algorithmic feedback loops, and fragmented cognitive processing (7, 8). In today's algorithmically mediated environments, individuals are increasingly experiencing symptom constellations that fall outside conventional diagnostic categories—necessitating an urgent reconceptualization of mental health nosology.

Among these emergent conditions are novel syndromes that exemplify the psychological impact of digital environments. Emerging syndromes such as Continuous Partial Attention Disorder (CPAD), Algorithm Dependency Disorder (ADD*)*, and Digital Anxiety Disorder (DAD) exemplify this shift, highlighting the urgent need for updated diagnostic categories that capture the intersection between digital behaviour and mental health (9–11). CPAD is characterized by chronic attentional fragmentation across competing digital streams; ADD by overreliance on algorithmic recommendations for decision-making; and DAD by anticipatory anxiety linked to digital metrics and notifications. These conditions reflect maladaptive adaptations to technology use that can compromise emotional regulation, executive functioning, and psychological resilience. Despite growing recognition of these syndromes, clinicians currently lack structured diagnostic frameworks or clinical guidelines to assess and address them. Therefore, a critical gap exists in equipping practitioners with tools to navigate this emerging terrain.

This study advances a novel diagnostic framework tailored to the digital era, aiming to equip clinicians—regardless of technological expertise—with the tools necessary to identify, assess, and intervene in cases of emerging digital psychopathologies. Drawing on insights from cognitive science, social psychology, and computational psychiatry, the proposed framework recognizes digital stimuli not as peripheral lifestyle factors but as central contributors to psychopathological processes (12, 13). Unlike traditional models, this framework conceptualizes digital stimuli as integral causal agents in mental health outcomes, offering operationalizable criteria for clinical practice. Two recent systematic reviews (14, 15) have emphasized the growing impact of digital hyperconnectivity on mental health symptomatology across global populations, reinforcing the need for reconceptualization.

**Figure 1:**
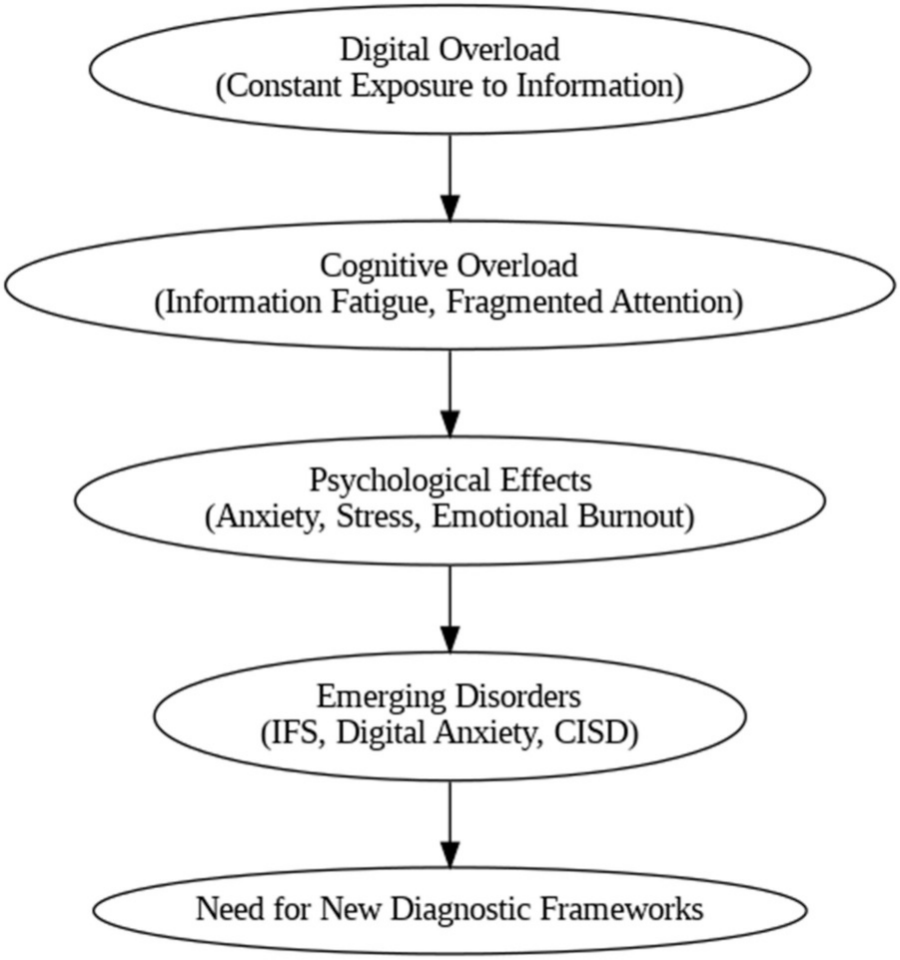
The psychological progression of digital overload.

Figure 1 illustrates the psychological trajectory of digital overload, mapping its progression from continuous exposure and cognitive fragmentation to psychological distress and emergent psychopathological syndromes.

**Figure 2:**
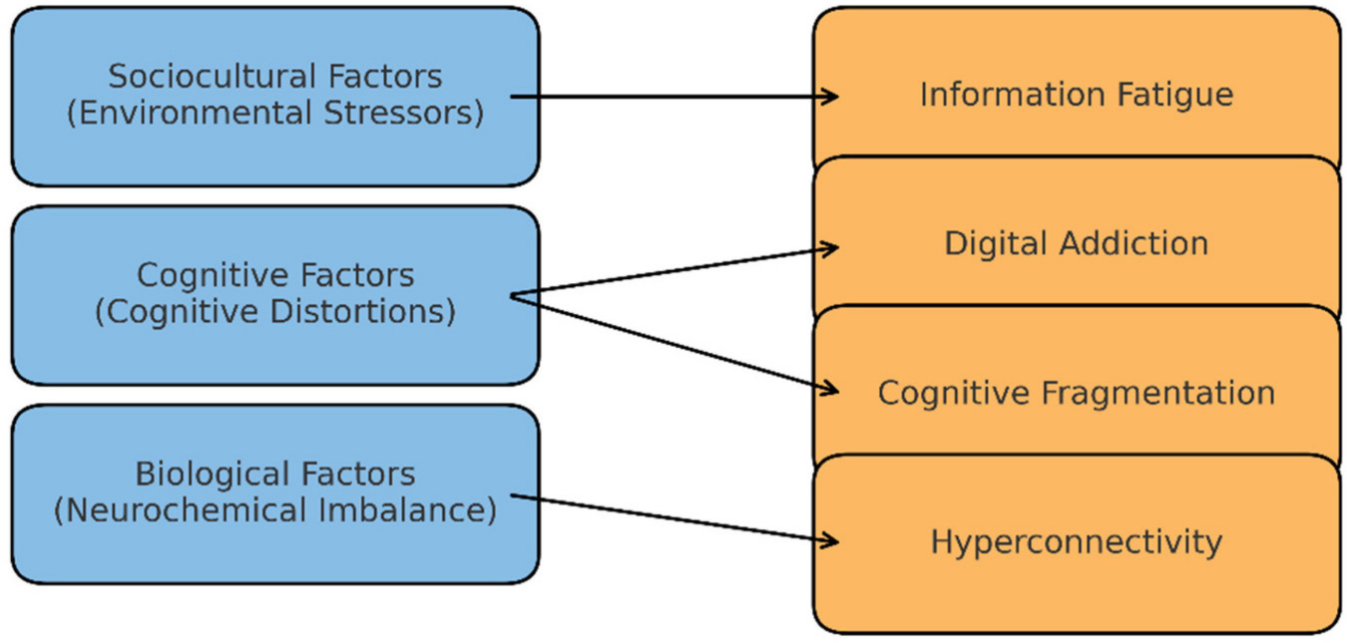
Shift in psychopathology: traditional to digital-era.

Figure 2 expands this model to demonstrate how traditional psychopathological variables—biological (e.g., neurochemical imbalance), cognitive (e.g., distortions), and sociocultural (e.g., environmental stressors)—now intersect with digital-era influences such as algorithmic reinforcement, digital fatigue, and attention switching (16, 17).

By proposing an updated paradigm for understanding digital-era psychopathology, this study lays the groundwork for a modernized mental health diagnostic system. In doing so, it seeks to enhance the clinical accuracy of assessments and empower practitioners to deliver interventions that restore cognitive clarity, emotional balance, and psychological resilience in a hyperconnected world (18, 19).

The following sections of this paper present the theoretical underpinnings, empirical support, and diagnostic structure for this emerging framework.

## 2 Theoretical foundations & conceptual framework

This paper has established a classification method for present-day psychological disorders by using established psychological theories which explain complete cognitive and emotional alterations due to prolonged digital media contact. Every classification category corresponds with specific theoretical structures showing the ways digital systems strengthen mental health difficulties.

Cognitive Fragmentation and Digital Overload Disorders are theoretically anchored in Cognitive Load Theory (20) and Flow Theory (21); social media and Immersive Technology-Induced Disorders are grounded in Social Comparison Theory (22) and Hyperpersonal Communication Theory (23); Technology Integration Disorders draw on Self-Determination Theory (82); and Symptom-Driven Disorders reference DSM-5 diagnostic criteria.

## 3 Visual representation of the proposed classification

The schematic shows four main classes of psychopathologies which organise distinctions based on cognitive, emotional, and behavioural disturbances of patients. The classes include the following:

▪ **Type A:** Cognitive Fragmentation and Digital Overload Disorders: caused by overexposure to information and saturation of mental capacity; therefore, they lead to deterioration in a person's attention and memory.
▪ **Type B:** Social media and Immersive Technology-Induced Disorders: Concerning disorders resulting from hyper-connectivity and the use of social media that enhance negative social comparisons and distorted self-concepts.
▪ **Type C:** Human autonomy together with critical thinking deteriorate when people depend too heavily on algorithms and Artificial Intelligence systems in their daily lives.
▪ **Type D:** The category of Symptom-Driven Disorders includes disorders which align with diagnostic criteria from DSM-5, while their progression depends significantly on continuous usage of digital technology.

It offers an all-inclusive framework through which all the different types of psychopathologies that occur in the digital era can be captured.

**Figure 3:**
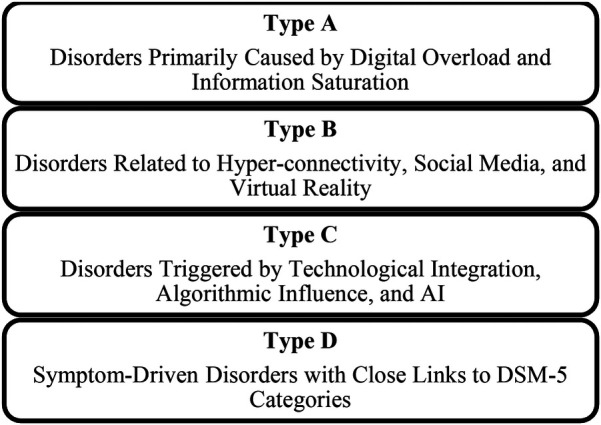
Classification framework for digital-Era psychopathologies.

As shown in Figure 3, The proposed classification is based on four different psychological frameworks, providing a foundation for the cognitive and emotional disruptions that arise from these digital environments. Thus, they provide the basis from which there can be structured approaches to diagnosing and addressing these mental health challenges.

### 3.1 Cognitive fragmentation and digital overload disorders

Disorders under this category, such as Continuous Partial Attention Disorder (CPAD) and Digital Dissociation Syndrome (DDS), result from excessively exciting digital stimuli that cause an individual's cognitive overload. Based on the Cognitive Load Theory proposed by Sweller in 1988 and the Flow Theory proposed by Csikszentmihalyi in 1990, this category describes how digital environments disrupt attention and memory and, consequently, degrade cognitive functioning (23) Recent applications of Flow Theory to digital gaming and attention disorders further support this classification (24). These disorders represent how split digital interactions affect brain functions because extended multitasking, alongside information overload, results in both cognitive exhaustion and isolation (25).

### 3.2 Disorders induced by social media and immersive technology

Type B disorders emerge from excessive social media use that enables people to compare themselves negatively to others and fail to perceive themselves accurately. A combination of Social Comparison Theory (Festinger, 22) and Hyper-personal Communication Theory (Walther, 23) explains the development of SMIND and DPD, since they manifest through digital interactions that produce self-importance exaggeration while dysregulating emotional responses (26). Recent research has expanded Social Comparison Theory's relevance to Instagram and TikTok-driven body image dissatisfaction (27). Algorithms raise the frequency of emotionally-charged content that enables emotional volatility and makes users develop compulsive behaviour until they get validation (28).

### 3.3 Disorders of technology integration

The extensive implementation of AI and algorithms creates two different disorders: ADD and AIDD, which reduce critical capabilities and self-autonomy through algorithmic rules. According to Self-Determination Theory (82), the principles explain that pathologies develop from intrinsic motivation and cognitive independence failure, which produces decision paralysis and identity confusion.

People develop a dependence on algorithmic suggestions through daily mediated decision-making with AI systems, which reduces their decision-making abilities along with cognitive engagement (8). The reliance on algorithms may stunt individual growth of self-directed choice abilities while weakening inner drive, since studies prove that algorithm-dependence interferes with personal planning and initiative (8). Alarmingly, studies establish that users who depend on algorithms experience reduced psychological well-being as their identities split apart, especially when they accept external algorithmic feedback as defining themselves (27–29).

### 3.4 Symptom-based disorders that require diagnostic adaptation

**Figure 4:**
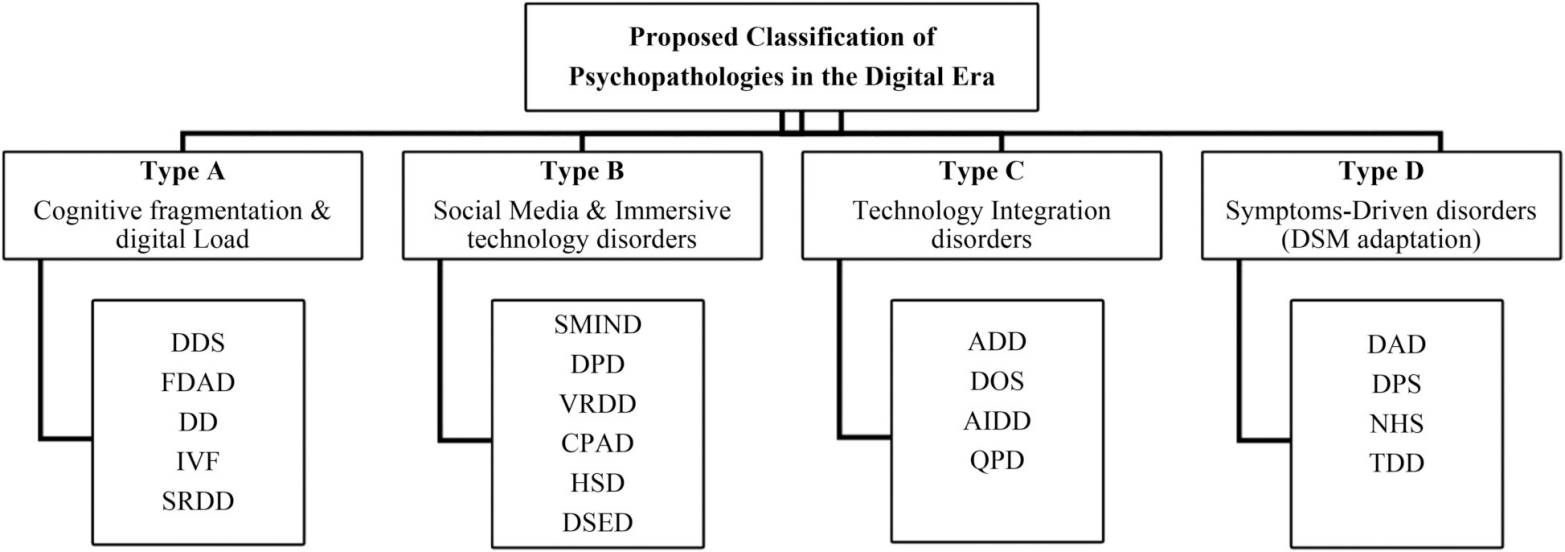
Visual taxonomy of emerging digital-Era psychopathologies.

These disorders reflect traditional DSM-5 categories but are specifically influenced by digital engagement. Digital Anxiety Disorder (DAD) and Temporal Dysregulation Disorder (TDD) represent examples of how continuous digital monitoring and hyper-connectivity contribute to increased anxiety and cognitive deficits. These disorders, even though they resemble traditional conditions such as GAD, are differentiated by their origins in digital environments, highlighting the need for adapted diagnostic criteria and treatments. The occurrence of these conditions demands that medical professionals reevaluate traditional diagnostic approaches. Digital technology use has transformed how symptoms appear as well as affected individual mental well-being conditions (12). Scientists now establish compulsive social media behaviour along with FOMO and online validation metrics as risk factors which produce stress-related conditions and unhealthy routines (15, 30). Digital overstimulation creates problems with emotional regulation and short attention span, which affect younger people according to Odgers & Jensen (83). Clinical professionals should use digital behaviour assessments during diagnostic interviews because these results demonstrate that healthcare practices need to integrate real-world digital realities into treatment strategies. Table 1 classifies emerging psychopathologies of the digital era based on their theoretical base to have a brief yet deep overview of those cognitive-emotional disruptions associated with hyper-connectivity. Table 1 & Figure 4 summarizes theoretical mappings for all categories; Table 2 expands diagnostic overlaps with DSM-5.

**Table 1:** Theoretical classification of emerging digital-era psychopathologies.

Category	Theoretical base	Key concepts	Disorders
Type A—Cognitive fragmentation and digital overload disorders	Cognitive Load Theory (20) and Theory of Flow (21)	▪ Cognitive Load Theory: Excessive digital stimuli overwhelm cognitive capacity, impairing memory, decision-making, and problem-solving. ▪ Theory of Flow: Disruptions in optimal focus due to digital distractions lead to frustration and decreased satisfaction.	▪ Continuous Partial Attention Disorder (CPAD) ▪ Cognitive Fragmentation
Type B—Social-media and immersive-technology-induced disorders	Social Comparison Theory (22) and Hyper-personal Communication Theory (23)	▪ Social Comparison Theory: Social media fosters negative self-perception through constant comparison with idealised lives. ▪ Hyper-personal Communication Theory: Lack of non-verbal cues in digital communication leads to distorted emotional responses.	▪ Social Media-Induced Narcissistic Disorder (SMIND) ▪ Digital Perfectionism Disorder (DPD) ▪ Digital Social Empathy Deficiency (DSED)
Type C—Technological-integration disorders	Self-Determination Theory (82)	▪ Loss of autonomy and reliance on algorithms in digital environments undermines intrinsic motivation and personal agency.	▪ Algorithm Dependency Disorder (ADD) ▪ Loss of Personal Agency
Type D—Symptom-driven disorders requiring diagnostic adaptation	Attentional Control Theory (Eysenck et al., 2007) and Cognitive Load Theory (20)	▪ Attentional Control Theory: Anxiety disrupts attentional focus, impairing cognitive efficiency. ▪ Cognitive Load Theory: Information overload in digital environments contributes to stress and emotional volatility.	▪ Digital Anxiety Disorder (DAD) ▪ Attention Deficit due to Anxiety

CPAD, continuous partial attention disorder; SMIND, social media-induced narcissistic disorder; DPD, digital perfectionism disorder; DSED, digital social empathy deficiency; ADD, algorithm dependency disorder; DAD, digital anxiety disorder.

**Table 2:** Comparative analysis of emerging digital-Age disorders and their relationship to DSM-5 diagnoses.

Type	Disorder name	Core symptoms	Unique digital features	Clinical relevance/interventions	DSM-5 disorder similarities	Key distinctions from DSM-5
A—Cognitive overload and fragmentation	Digital Dissociation Syndrome (DDS)	Emotional detachment; depersonalization; derealization	Disconnection between online and offline selves	Develop diagnostic tools addressing online–offline identity dissonance	Depersonalization/Derealization Disorder	DDS arises from *digital identity disconnect* rather than intrinsic detachment from reality
	FOMO-Driven Anxiety Disorder (FDAD)	Anxiety, social comparison, sleep disruption	Obsessive digital monitoring to avoid missing trends	Cognitive–behavioral therapy to reduce social-comparison anxiety	Generalized Anxiety Disorder (GAD)	FDAD characterized by *digital monitoring–related anxiety*, absent in GAD
	Doomscrolling Disorder (DD)	Anxiety, hopelessness, insomnia	Compulsive consumption of negative online content	Encourage balanced media consumption and positive routines	Major Depressive Disorder (MDD)	DD centers on *compulsive exposure to negative digital content*
	Information Validation Fatigue (IVF)	Cognitive exhaustion; decision paralysis	Overchecking and skepticism from constant fact-verification	Psychoeducation on balanced information engagement	Generalized Anxiety Disorder (GAD)	IVF reflects *digital fact-checking exhaustion*, not generalized worry
	Synthetic Reality Detachment Disorder (SRDD)	Existential crisis; detachment from reality	Preference for synthetic or augmented environments	Therapy to re-establish real-world identity continuity	Depersonalization/Derealization Disorder	SRDD specific to *synthetic-reality immersion*
B—Social-media and immersive-technology disorders	Social Media-Induced Narcissistic Disorder (SMIND)	Compulsive validation seeking; inflated self-image	Reliance on social feedback metrics	Tailored therapy addressing social-media dependence	Narcissistic Personality Disorder (NPD)	SMIND driven by *platform-based validation*, unlike generalized NPD
	Virtual Reality Dependence Disorder (VRDD)	Social isolation; neglect of real-world roles	Preference for immersive virtual spaces	Reduce VR exposure; promote real-world reintegration	Avoidant Personality Disorder	VRDD = avoidance through virtual immersion; Avoidant PD = interpersonal withdrawal
	Continuous Partial Attention Disorder (CPAD)	Cognitive fog; distractibility; restlessness	Self-induced multitasking in digital settings	Attention-training and digital-focus interventions	Attention-Deficit/Hyperactivity Disorder (ADHD)	CPAD = *contextual multitasking overload*; ADHD = neurodevelopmental origin
	Hyperconnectivity Stress Disorder (HSD)	Chronic stress; irritability; burnout	Pressure to remain constantly available online	Digital-boundary setting and mindfulness	Generalized Anxiety Disorder (GAD)	HSD = stress from *connectivity expectations*, absent in GAD
	Digital Perfectionism Disorder (DPD)	Unrealistic self-standards; body dysmorphia	Compulsive editing and curation of online persona	Cognitive restructuring of self-image standards	Body Dysmorphic Disorder (BDD)	DPD = *digital-image curation focus*; BDD = physical appearance fixation
	Digital Social Empathy Deficiency (DSED)	Reduced empathy; emotional indifference	Limited affect due to absence of non-verbal cues	Empathy-training therapy and social-skills restoration	Antisocial Personality Disorder (ASPD)	DSED = *empathy erosion* via *digital mediation*; ASPD = broader antisociality
C—Technological-integration and dependency disorders	Algorithmic Dependency Disorder (ADD)	Reduced critical thinking; indecision	Overreliance on algorithmic outputs	Promote independent decision-making	Obsessive–Compulsive Disorder (OCD)	ADD = *externally guided compulsivity*; OCD = *internally driven obsessions*
	Data Obsession Syndrome (DOS)	Anxiety over data loss; information hoarding	Compulsive accumulation of digital content	Behavioral therapy targeting data-hoarding anxiety	Obsessive–Compulsive Disorder (OCD)	DOS = *digital hoarding behavior*; OCD = *ritualistic fear-avoidance*
	AI Identity Diffusion Disorder (AIDD)	Identity confusion; existential unease	Overidentification with AI systems and outputs	Strengthen self-concept and human-agency boundaries	Personality-related Identity Disturbance	AIDD = *AI-mediated self-diffusion*, not internal instability
	Quantum Paranoia Disorder (QPD)	Paranoid ideation; hypervigilance	Fear of quantum technologies compromising security	Psychoeducation to reduce future-tech anxiety	Paranoid Personality Disorder (PPD)	QPD = *technology-future paranoia*; PPD = interpersonal mistrust
D—Anxiety, vigilance, and temporal dysregulation	Digital Paranoia Syndrome (DPS)	Hypervigilance; compulsive account deletion	Fear of surveillance and privacy invasion	CBT for rational risk appraisal	Paranoid Personality Disorder (PPD)	DPS = *digital-surveillance fear*, absent in PPD
	Notification Hypervigilance Syndrome (NHS)	Anxiety; sleep disturbance; hyperarousal	Overreaction to notification alerts	Mindfulness and digital-detox training	Post-Traumatic Stress Disorder (PTSD)	NHS = *alert-triggered anxiety*, distinct from trauma exposure
	Temporal Dysregulation Disorder (TDD)	Distorted time perception; procrastination	Digital overuse disrupts temporal awareness	Time-management and CBT interventions	Circadian Rhythm Disorders	TDD = *perceived time distortion*, not physiological
	Digital Anxiety Disorder (DAD)	Heightened anxiety; compulsive monitoring	Triggered by sustained social-media engagement	Reduce digital dependency and anxiety behaviors	Generalized Anxiety Disorder (GAD)	DAD = *technology-induced anxiety*, unlike generalized worry

DDS, digital dissociation syndrome; FDAD, FOMO-driven anxiety disorder; DD, doomscrolling disorder; IVF, information validation fatigue; SRDD, synthetic reality detachment disorder; SMIND, social media-induced narcissistic disorder; VRDD, virtual reality dependence disorder; CPAD, continuous partial attention disorder; HSD, hyperconnectivity stress disorder; DPD, digital perfectionism disorder; DSED, digital social empathy deficiency; ADD, algorithmic dependency disorder; DOS, data obsession syndrome; AIDD, AI identity diffusion disorder; QPD, quantum paranoia disorder; DPS, digital paranoia syndrome; NHS, notification hypervigilance syndrome; TDD, temporal dysregulation disorder; DAD, digital anxiety disorder.

Table 2 analyses the characteristics of each disorder in detail, hence giving insight into its clinical relevance and resemblance to the DSM-5 disorders. Symptoms of DDS include emotional dissociation associated with dissonance arising between online and offline identities, much like Depersonalization/Derealization Disorder, but this time in a digital setting. Similarly, ADD also shares characteristics with OCD, but the compulsions come from outside algorithms. This perspective calls for new diagnostic tools since the digital influence on mental health is changing fast. The table fills in the gap between the traditional diagnostic frameworks and the emerging psychopathologies of the digital era.

#### 3.4.1 Empirical validation of emerging digital-era psychopathologies

The pervasive integration of digital technologies into daily life has catalyzed the emergence of novel mental health syndromes—psychopathologies that extend beyond the scope of traditional diagnostic systems. Increasingly, these digital-era conditions are being documented in contemporary literature and acknowledged in clinical settings, necessitating rigorous empirical validation to support their conceptual legitimacy and clinical applicability.

#### 3.4.2 Evidence-based validation

Empirical validation is essential to establish the clinical relevance of emerging digital psychopathologies and to support the development of accurate, evidence-based diagnostic and therapeutic frameworks (31). Recent studies underscore the profound psychological effects of sustained digital engagement. For instance, Elhai et al. (32) identified significant associations between excessive smartphone use and elevated anxiety and depressive symptoms—findings that align closely with the symptomatology of FOMO-Driven Anxiety Disorder (FDAD). Similarly, Montag et al. (33) employed neuroscientific methodologies to examine the impact of social media on emotional regulation and personality traits, lending credence to the conceptualization of Social Media-Induced Narcissistic Disorder (SMIND).

Emotional dysregulation, a hallmark of Digital Perfectionism Disorder (DPD), has also been substantiated through the work of Bányai et al. (34), who investigated the adverse effects of problematic Facebook use. Furthermore, Tarafdar et al. (35) introduced the construct of “technostress” as a foundational mechanism underlying Hyperconnectivity Stress Disorder (HSD), reinforcing the need for disorder-specific diagnostic constructs.

More recent evidence strengthens these associations. Studies show that problematic social networking use is significantly linked with anxiety and depression in adolescents and young adults, mediated by digital addiction and self-esteem dysregulation (36–41). Distorted cognition on social media platforms has also been empirically tied to underlying affective disorders (42). These findings support the need for disorder-specific interventions.

Collectively, these findings provide a growing body of empirical support for the classification of digital-era psychopathologies and highlight the urgency of developing structured diagnostic criteria, validated assessment instruments, and tailored intervention models for this emerging domain.

#### 3.4.3 Thematic classification

To enhance diagnostic clarity and clinical utility, the 19 identified digital-era psychopathologies have been organized into four thematic domains, as illustrated in Table 3, each reflecting a distinct cluster of psychological disturbances.

**Table 3:** Thematic classification of digital-Era psychopathologies.

Thematic domain	Disorder
Cognitive-Attentional Dysregulation	Continuous Partial Attention Disorder (CPAD)
	Temporal Dysregulation Disorder (TDD)
	Information Validation Fatigue (IVF)
Affective and Anxiety Disorders	Digital Anxiety Disorder (DAD)
	Doomscrolling Disorder (DD)
	FOMO-Driven Anxiety Disorder (FDAD)
	Hyperconnectivity Stress Disorder (HSD)
Identity and Social Disorders	Digital Dissociation Syndrome (DDS)
	Social Media-Induced Narcissistic Disorder (SMIND)
	AI Identity Diffusion Disorder (AIDD)
	Digital Social Empathy Deficiency (DSED)
	Synthetic Reality Detachment Disorder (SRDD)
Behavioral Addictions and Dependencies	Algorithmic Dependency Disorder (ADD)
	Virtual Reality Dependence Disorder (VRDD)
	Notification Hypervigilance Syndrome (NHS)
	Quantum Paranoia Disorder (QPD)
	Digital Paranoia Syndrome (DPS)
	Data Obsession Syndrome (DOS)
	Digital Reward Loop Addiction (DRLA)

Each disorder is characterized by a unique symptom profile, grounded in both theoretical constructs and empirical findings from contemporary psychological and psychiatric research.

Table 4 presents a cross-classification of the 19 proposed digital-era psychopathologies, illustrating their functional categorization based on typological classification (Type A–D) and their clinical grouping within thematic domains. The typology captures the underlying psychological mechanisms, while the thematic domains reflect observable clinical symptomatology.

**Table 4:** Typology vs thematic classification matrix.

Disorder	Typological classification	Thematic domain
Continuous Partial Attention Disorder (CPAD)	A	Cognitive-Attentional Dysregulation
Temporal Dysregulation Disorder (TDD)		
Information Validation Fatigue (IVF)		
Digital Anxiety Disorder (DAD)	B	Affective and Anxiety Disorders
Doomscrolling Disorder (DD)		
FOMO-Driven Anxiety Disorder (FDAD)		
Hyperconnectivity Stress Disorder (HSD)		
Digital Dissociation Syndrome (DDS)	C	Identity and Social Disorders
Social Media-Induced Narcissistic Disorder (SMIND)		
AI Identity Diffusion Disorder (AIDD)		
Digital Social Empathy Deficiency (DSED)		
Synthetic Reality Detachment Disorder (SRDD)		
Algorithmic Dependency Disorder (ADD)	D	Behavioral Addictions and Dependencies
Virtual Reality Dependence Disorder (VRDD)		
Notification Hypervigilance Syndrome (NHS)		
Quantum Paranoia Disorder (QPD)		
Digital Paranoia Syndrome (DPS)		
Data Obsession Syndrome (DOS)		
Digital Reward Loop Addiction (DRLA)		

Typology = underlying psychological mechanism; Thematic domain = observed symptom cluster.

This typological system (Types A–D) enables symptom-based differentiation grounded in psychological mechanisms (Table 2). Each disorder is mapped to both a diagnostic type and clinical domain, as shown in Table 1. The interaction between functional typology and clinical classification is visualized in Figure 1, highlighting the model's multidimensional applicability.

#### 3.4.4 Empirical Synthesis

A synthesis of empirical evidence published between 2020 and 2024 reinforces the clinical validity of the proposed conditions:

- Significant statistical correlations have been observed between digital usage patterns and the manifestation of anxiety-related and affective symptoms, including those characteristic of FDAD, DD, and DAD (43).
- Neurocognitive impairments related to multitasking, attentional fragmentation, and information overload have been linked to disorders such as CPAD and IVF (44–46).
- Identity diffusion and psychological detachment, particularly within immersive digital environments (e.g., social media platforms, AI systems, and virtual reality), are increasingly associated with DDS, AIDD, and VRDD (47).
- Heightened anxiety responses tied to surveillance, data privacy concerns, and digital overstimulation are symptomatic of disorders like Quantum Paranoia Disorder (QPD) and Digital Paranoia Syndrome (DPS) (48, 49).

These converging lines of evidence underscore the pressing need to revise existing psychiatric taxonomies to reflect the evolving psychological realities of the digital era (50). They also provide a foundation for advancing digital phenotyping, refining diagnostic tools, and guiding the development of targeted interventions.

Building on the empirical themes presented above, Table 5 presents concise diagnostic profiles for each of the 19 conceptualized digital-era psychopathologies. These disorders are further organized in Table 5, classified according to both their underlying mechanism (Type A–D) and their thematic domain.

**Table 5:** Empirical summary table of digital-Era psychopathologies.

Disorder	Typology	Thematic domain	Empirical focus
Continuous Partial Attention Disorder (CPAD)	A	Cognitive-Attentional Dysregulation	Cognitive overload from digital multitasking (51)
Temporal Dysregulation Disorder (TDD)			Distorted time perception due to digital immersion (52)
Information Validation Fatigue (IVF)			Fatigue from compulsive digital fact-checking (53)
FOMO-Driven Anxiety Disorder (FDAD)	B	Affective and Anxiety Disorders	Anxiety from fear of missing out and social comparison (54)
Doomscrolling Disorder (DD)			Addiction to negative digital news cycles (55)
Digital Anxiety Disorder (DAD)			Persistent anxiety from digital metrics and stimuli
Hyperconnectivity Stress Disorder (HSD)			Burnout from sustained digital connectivity (56)
Digital Dissociation Syndrome (DDS)	C	Identity and Social Disorders	Identity splitting across online and offline selves (57)
Social Media-Induced Narcissistic Disorder (SMIND)			Narcissistic behaviors reinforced via social media validation (58)
AI Identity Diffusion Disorder (AIDD)			Self-concept confusion induced by AI interaction (59)
Digital Social Empathy Deficiency (DSED)			Lack of empathy and poor perspective-taking online (60)
Synthetic Reality Detachment Disorder (SRDD)			Preference for virtual over physical reality (61)
Algorithmic Dependency Disorder (ADD)	D	Behavioral Addictions and Dependencies	Dependence on algorithmic decision-making (62)
Virtual Reality Dependence Disorder (VRDD)			Avoidance and withdrawal into virtual reality (63)
Notification Hypervigilance Syndrome (NHS)			Hyper-alertness to notifications and digital cues (64)
Digital Reward Loop Addiction (DRLA)			Addiction to reward-driven digital engagement (65)
Quantum Paranoia Disorder (QPD)			Anxiety from surveillance and data insecurity (66)
Digital Paranoia Syndrome (DPS)			Behavioral change due to perceived surveillance (67)
Data Obsession Syndrome (DOS)			Obsessive concern with digital data control and loss (68)

Note: Emerging AI–related disorders (e.g., Algorithmic Dependency Disorder, AI Identity Diffusion Disorder) are conceptually informed by current scholarship on AI ethics, fairness, and policy frameworks [78–81].

## 4 Visual overview of diagnostic process

The diagnostic flowcharts represent a new structured way for clinicians to identify and categorise psychopathologies that have only now emerged associated with the digital world. These diagnostic instruments enable easy diagnosis by narrowing down symptoms related to digital overload, hyper-connectivity, and algorithm dependency so that mental health professionals can map symptoms onto four emerging disorder categories. The flow diagrams guide the clinician systematically from a broad spectrum to symptom identification, and such diagnoses ensure that the complexities of mental health problems in the digital era are checked and filtered for proper intervention.

**Figure 5:**
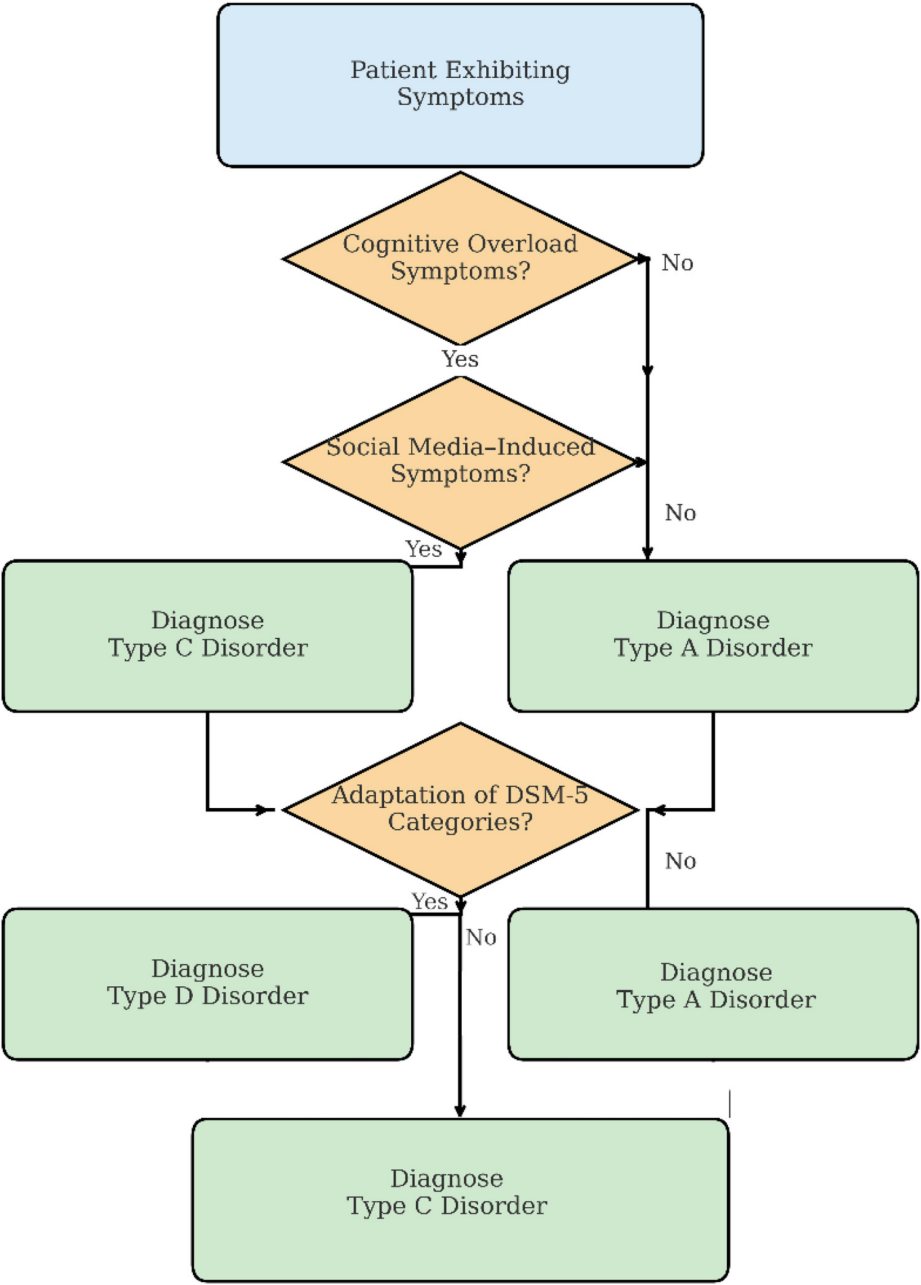
Diagnostic flowchart for emerging digital-era psychopathologies. The diagram progresses top-to-bottom, beginning with presenting symptoms, with affirmative (“Yes”) decisions directing the flow downward and negative (“No”) decisions branching rightward, illustrating the stepwise diagnostic pathway from initial symptom assessment through category assignment.

This flowchart in Figure 5 offers an organized framework for clinicians to categorize symptom clusters into one of four proposed psychopathological types (A–D), which include digital overload, hyperconnectivity, algorithmic dependency, and digital-contextual variants of DSM-5 disorders. The structure aids in planning and identification by aligning the presenting symptoms with the corresponding digital age typology.

▪ Initial Symptom Verification: The diagnostic evaluation commences with analyzing the symptoms of cognitive fragmentation and attentional deficits—indicative of “A”-Type disorders like Continuous Partial Attention Disorder (CPAD). These symptoms manifest due to excessive digital environment, multitasking, and information saturation.
▪ Evaluation of Social-Mediated Dysregulation: This afflictive node concerns the affective symptoms stemming from social media use. Type B disorders, like Social Media Induced Narcissistic Disorder SMIND, and FOMO-Driven Anxiety Disorder (FDAD), exhibit compulsive validation-seeking and emotional dysregulation due to hyper-connectivity.
▪ Recognition of Algorithmic Dependence: With decreased self-agency and increased reliance on digital mechanisms for decisions as self-automation frameworks, the clinician thinks of the Type C range Algorithmic Dependency Disorder (ADD) and similar afflictions. Select syndromes may need particular treatments (Algorithmic Recalibration Therapy) aimed at autonomy, intrinsic decision-making recovery, and dependence restoration.
▪ DSM-Type D Symptom Clusters: If the symptom profile corresponds with the more classical diagnoses of anxiety, paranoia, or even compulsive action, but appears to be aggravated by some or all digital conditions, then Type D disorders apply. Digital anxiety disorder (DAD) and digital paranoia syndrome (DPS) serve as primary exemplars of this hybrid class and thus require modified DSM digital context factors, which go beyond traditional contextual determinants.

## 5 Diagnostic and intervention framework for digital-era psychopathologies

### 5.1 Diagnostic flow logic

As illustrated in Figure 5, the proposed diagnostic flowchart aims to help clinicians classify symptom sets into one of four typological types (A–D), which are defined by the patient's disruption of the cognitive, affective, behavioral, or volitional domains.

Initial Symptom Identification. The diagnostic process begins with the evaluation of cognitive overload symptoms—hallmarks of Type A disorders, such as *Continuous Partial Attention Disorder (CPAD)*. These symptoms reflect fragmentation of attention, executive fatigue, and multitasking-related impairments.

Social-Mediated Dysregulation. If cognitive overload is not primary, the diagnostic flow proceeds to identifying symptoms associated with social media use—indicative of Type B disorders, such as *Social Media-Induced Narcissistic Disorder (SMIND)* or *FOMO-Driven Anxiety Disorder (FDAD)*. These disorders are characterized by emotional dysregulation, compulsive validation-seeking, and hyperconnectivity.

Algorithmic Dependence and Identity Erosion. The third diagnostic stage examines the degree of reliance on digital systems and algorithmic agents. Type C disorders, such as *Algorithmic Dependency Disorder (ADD)* or *AI Identity Diffusion Disorder (AIDD)*, emerge when decision-making autonomy and identity coherence are compromised by prolonged AI interaction. Interventions like Algorithm Recalibration Therapy may be appropriate here.

DSM-Analogous Symptomatology. If no clear digital trigger is apparent, yet the symptoms resemble traditional diagnoses (e.g., anxiety, paranoia), the diagnostic path proceeds to Type D disorders. These include *Digital Anxiety Disorder (DAD)* and *Digital Paranoia Syndrome (DPS)*, where classical symptoms are digitally mediated or amplified.

**Figure 6:**
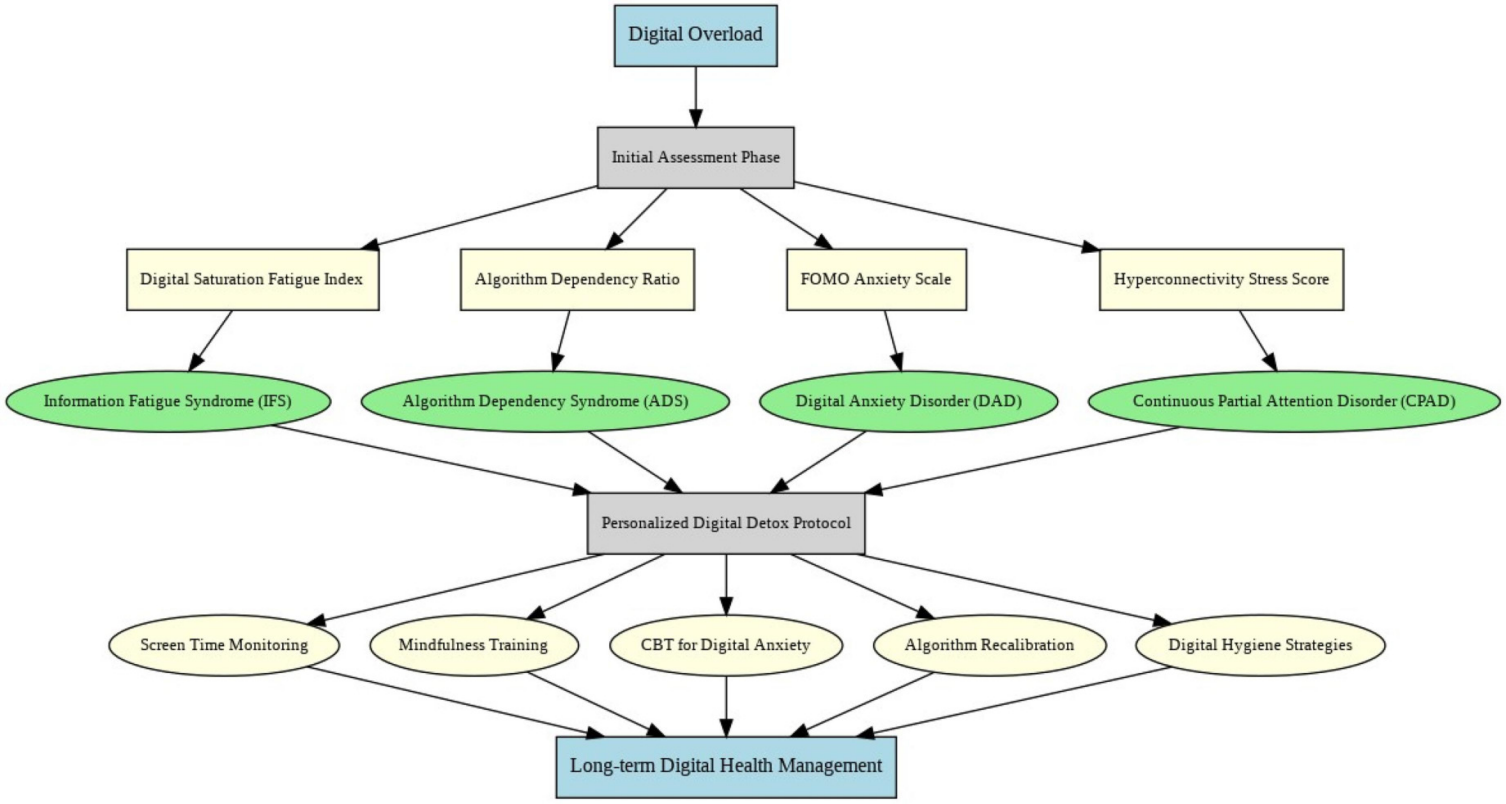
Diagnostic and intervention model for managing digital overload disorders. The framework is organized top-to-bottom, with patient symptoms initiating the flow, successive decision nodes separating cognitive, social, algorithmic, and DSM-analogous disturbances, and arrows showing the progressive logic by which clinicians classify cases into Types A–D and select corresponding intervention strategies.

As illustrated in Figure 6, This flowchart aids clinicians in guiding them through a stepwise classification approach aligned with core digital symptom domains which correlate with disorder type classifications (Types A–D) (69).

### 5.2 Quantitative diagnostic metrics

To operationalize the diagnostic process, this framework introduces four novel metrics—each derived from research in technostress, digital behavior, and cognitive overload. These instruments transform subjective digital experiences into quantifiable, diagnostic insights.

#### 5.2.1 Digital saturation fatigue index (DSFI)

The DSFI provides an objective measure of cognitive exhaustion resulting from prolonged digital engagement. It aggregates data on attention span, task-switching fatigue, and reaction time variance. High DSFI scores support diagnoses of *CPAD*, *IVF*, or *Doomscrolling Disorder (DD)* (70, 71).

#### 5.2.2 Algorithm dependency ratio (ADR)

The ADR quantifies the extent of reliance on algorithmic systems in daily decision-making (e.g., recommendations, navigation, content selection). Elevated scores suggest *Algorithmic Dependency Disorder (ADD)* or *AI Identity Diffusion Disorder (AIDD)* and reflect diminished critical autonomy (72, 73).

#### 5.2.3 FOMO anxiety scale (FAS)

Adapted from Przybylski et al. (52), the FAS evaluates compulsive checking, social comparison, and notification-driven anxiety. It integrates both psychometric and physiological indicators (e.g., HRV under notification load). High FAS scores point to *FDAD* and *Notification Hypervigilance Syndrome (NHS)* (6, 74).

#### 5.2.4 Hyperconnectivity stress score (HSS)

The HSS captures the psychological cost of persistent digital exposure. It combines subjective stress indices with objective measures such as sleep disruption, cortisol variability, and multitasking burden. It is used primarily in assessing *Hyperconnectivity Stress Disorder (HSD)* (75, 76).

### 5.3 Personalized digital detox protocol (PDDP)

Once a disorder is diagnosed, the clinician is guided to a personalized treatment track within the PDDP system. Each track aligns with the neurocognitive and behavioral signatures of the respective disorder (77).

Table 6 provides a comprehensive mapping of disorder-specific intervention protocols within the Personalized Digital Detox Protocol (PDDP). Each intervention is aligned with the typological classification (Types A–D) and grounded in the underlying symptom mechanisms of the disorder, enabling precision-targeted treatment pathways across the digital psychopathology spectrum.

**Table 6:** Personalized intervention mapping for digital-Era psychopathologies.

Disorder	Typology	Primary intervention
CPAD (Continuous Partial Attention Disorder)	A	Attention Restoration Therapy; Structured Digital-Offline Intervals
IVF (Information Validation Fatigue)		Cognitive Load Management; News Exposure Limitation
TDD (Temporal Dysregulation Disorder)		Time Awareness Training; Sleep Hygiene Protocol
DD (Doomscrolling Disorder)	B	Emotion Regulation CBT; News Curation Strategies
FDAD (FOMO-Driven Anxiety Disorder)		CBT; Notification Management; Social Comparison Reframing
DAD (Digital Anxiety Disorder)		Exposure Therapy; Mindfulness; Digital Psychoeducation
HSD (Hyperconnectivity Stress Disorder)		Stress Regulation Therapy; Multitasking Reduction Plan
SMIND (Social Media-Induced Narcissistic Disorder)	C	Self-Concept Integration Therapy; Validation Reframing
AIDD (AI Identity Diffusion Disorder)		Identity Consolidation Interventions; Reflective Dialogue with AI Narratives
DSED (Digital Social Empathy Deficiency)		Empathy Training; Perspective-Taking Tasks
SRDD (Synthetic Reality Detachment Disorder)		Reality Orientation CBT; Balanced Virtual/Physical Engagement Routines
ADD (Algorithmic Dependency Disorder)		Algorithm Recalibration Therapy (ART); Decision-Making Autonomy Exercises
VRDD (Virtual Reality Dependence Disorder)	D	Behavioral Activation; Controlled Immersion Schedule
NHS (Notification Hypervigilance Syndrome)		Notification Exposure Therapy; Stimulus Desensitization
QPD (Quantum Paranoia Disorder)		CBT for Digital Surveillance Anxiety; Security Education
DPS (Digital Paranoia Syndrome)		Exposure and Response Prevention; Online Behavior Restructuring
DOS (Data Obsession Syndrome)		OCD-Informed Digital Hoarding Therapy; Archival Reappraisal Techniques
DPD (Digital Perfectionism Disorder)		ACT for Self-Criticism; Social Media Realism Interventions

**Figure 7:**
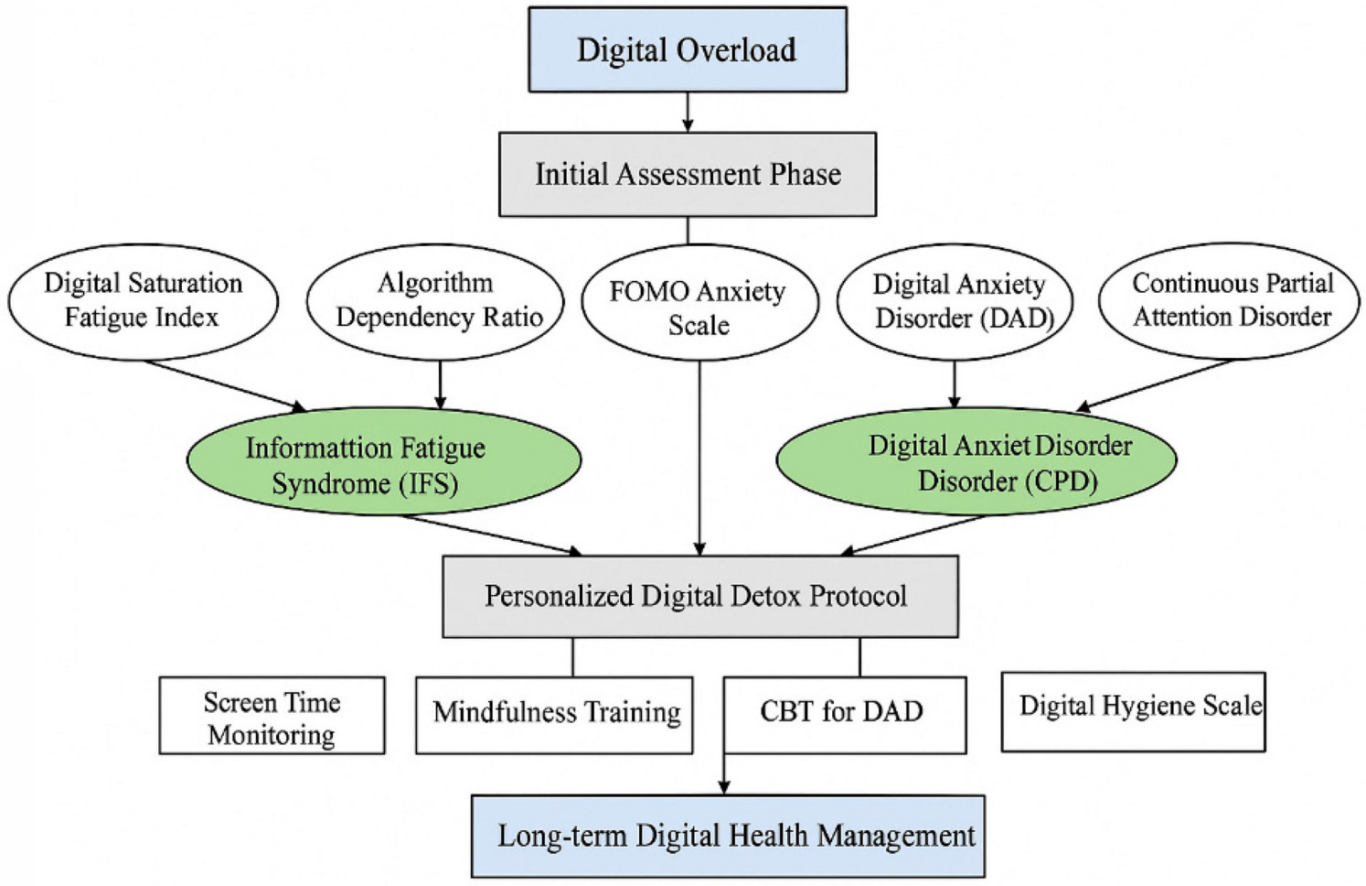
Integrated diagnostic and intervention model for managing digital-era psychopathologies. The model flows top-to-bottom, beginning with digital overload, advancing through assessment and disorder-specific identification, and guiding clinicians toward precision-tailored interventions within the Personalized Digital Detox Protocol, culminating in sustained digital health management.

This model, in Figure 7, maps disorder-specific profiles to precision diagnostics and targeted interventions, culminating in long-term digital health monitoring.

#### 5.3.1 Long-term digital hygiene management (LDHM)

All intervention pathways converge into the **LDHM phase**, during which patients undergo periodic reassessment using the DSFI, ADR, FAS, and HSS to monitor progress and prevent relapse. This ensures ongoing alignment between clinical intensity and patient need while promoting sustainable digital wellbeing.

## 6 Methodology

### 6.1 Study design

This cross-sectional pilot study was conducted between January and April 2025 to preliminarily assess the clinical recognizability, symptom coherence, and diagnostic plausibility of 19 newly conceptualized digital-era psychopathologies. The objective was to gather observational insights from licensed mental health professionals regarding their encounters with these disorders in clinical practice. The sample size (*N* = 75) was determined based on feasibility and pilot study design principles, recognizing the exploratory nature of the study and its aim to inform subsequent large-scale validation.

### 6.2 Participants

Purposive sampling of 75 licensed clinicians was conducted from psychiatric hospitals, private psychiatric clinics, mental health associations, and professional networks across the United Arab Emirates. To qualify, participant clinicians were required to (a) be issued a license in psychiatry, clinical psychology, or clinical social work, and (b) possess no less than two years of independent post-licensure clinical practice.

Out of 100 clinicians who were invited, 75 completed the survey, achieving a participation rate of 75%. The final sample comprised 48% clinical psychologists (*n* = 36), 40% psychiatrists (*n* = 30), and 12% clinical social workers (*n* = 9) indicating a slight majority in psychology. Participation was voluntary and anonymous under the institution's ethical approval framework.

### 6.3 Survey instrument

A digital-era psychopathology survey questionnaire tailored to assess clinician's recognition paradigms of the digital-era psychopathology was created by the researcher. Due to the new constructs, there were no instruments available. Thus, the new assessment tools were formulated, and verified by before the subject matter experts.

Each participant received standardized operational definitions for all 19 disorders. For each item, clinicians were asked to:

▪ Indicate whether they had encountered cases matching the definition (Yes/No)
▪ Rate the typical symptom severity (Mild, Moderate, Severe)
▪ Report diagnostic confidence (High, Moderate, Low)

To establish internal consistency, the instrument was piloted among 10 clinicians. As shown in Table 7 The Disorder Recognition Ratings section yielded strong reliability, with Cronbach's alpha = 0.87.

**Table 7:** Internal consistency of survey instrument (pilot sample, *N* = 10).

Section of survey	Number of items	Cronbach's alpha
Section 2: Disorder Recognition Ratings	19	0.87

### 6.4 Survey procedure

The final survey was conducted electronically via a secure, restricted-access platform. Completion of the survey was considered as consenting to participate in the study. No demographic information that can be used to trace the identity of the clinicians or the patients was collected.

Before pilot administration, the instrument was first evaluated for content validity by three senior clinical psychologists specializing in digital mental health. Suggestions were made by the experts regarding the operational definitions of the items, and their implementation increased specificity. High inter-rater reliability among the reviewers was noted (*κ* = 0.82, Cohen's kappa).

### 6.5 Patient chart review

To complement survey findings with clinical records, a retrospective chart review of 225 adult patient files was conducted. Charts were selected via stratified random sampling proportional to caseload size and care setting. Inclusion required that patients were aged 18 or older and received treatment within the previous 12 months.

Data were extracted using a standardized Patient Chart Checklist developed for this study, capturing:

▪ Documented symptomatology consistent with each disorder (✓/×)
▪ Symptom severity (Mild, Moderate, Severe)
▪ Clinician-rated diagnostic confidence (High, Moderate, Low)

To ensure reliability, 10% of charts (*n* = 23) were double-coded independently. Agreement between coders was substantial (*κ* = 0.80). Chart reviewers were blinded to their own survey responses to reduce bias.

### 6.6 Data analysis

All analyses were conducted using the IBM SPSS Statistics for Windows, Version 29.0 (IBM Corp., Armonk, NY, USA). Before detailed analysis, the dataset underwent a screening process for missing values and outliers. For incomplete responses, listwise deletion was utilized. The dataset's continuous variables underwent the Shapiro–Wilk test for normal distribution; no significant violations were found which meant nonparametric methods could be used due to the ordinal and categorical nature of the variables.

### 6.7 Clinician survey data

Descriptive and inferential statistical analyses were employed to explore clinicians' recognition patterns of digital-era psychopathologies. The following steps were applied:

- **Prevalence Estimation**: For each of the 19 disorders, prevalence was calculated as the proportion of clinicians who endorsed having observed symptomatology consistent with the operational definition.
- **Severity and Diagnostic Confidence**: Clinician-reported symptom severity (categorized as *mild*, *moderate*, or *severe*) and diagnostic confidence (rated as *high*, *moderate*, or *low*) were analyzed using frequency distributions and percentages.
- **Confidence Intervals**: 95% confidence intervals (CIs) for the observed prevalence rates were calculated using the standard binomial formula, enhancing interpretability and precision of estimates:

Equation 1. Binomial Confidence Interval Formula.

$$CI_{\left\{95\text{\%}\right\}}=\;p\;\pm 1.96\;\sqrt{\left\{\frac{\;p(1\;-\;p)}{n}\right\}}$$ (1)

where *p^* is the observed proportion, *n* is the sample size (*N* *=* *75*), and *z* is the standard normal value corresponding to the desired confidence level (for a 95% CI, *z* *=* *1.96*).

**Inferential Analysis**: Nonparametric inferential tests were applied exclusively to the survey data due to the ordinal structure and sample size:

- **Chi-square tests** were used to examine associations between clinician characteristics (e.g., profession, years of experience) and disorder recognition.
- **Spearman's rank-order correlations** were calculated to explore associations between perceived disorder severity and diagnostic confidence.

No parametric analyses were conducted to avoid assumption violations given the categorical data and pilot sample size. Results were interpreted conservatively in light of the exploratory study design.

### 6.8 Patient chart review data

The chart review data were analyzed descriptively to summarize documented symptomatology consistent with the 19 digital-era disorders:

- **Symptom Presence**: Frequencies and proportions were computed for each disorder based on clinician indication (✓/×) of its presence in the patient's chart.
- **Symptom Severity and Confidence**: Extracted ratings of severity and diagnostic confidence were summarized using categorical frequency distributions.
- **Prevalent Patterns**: Particular attention was given to the most frequently documented disorders, including *Continuous Partial Attention Disorder (CPAD)*, *Digital Anxiety Disorder (DAD)*, and *Doomscrolling Disorder (DD)*, to identify preliminary clinical patterns.

Inferential statistical testing was not applied to the chart review data. This was due to the retrospective design, limited sample structure, and heterogeneity in clinical documentation practices that precluded valid assumption testing. The chart review served primarily to provide descriptive, real-world support for the symptomatology reflected in the survey data.

## 7 Ethical considerations

Ethical approval for the chart review was obtained from the Research Ethics Issues Committee (REIC) at Al Ain University. Formal IRB review for the clinician survey was waived due to its anonymous, minimal-risk design. All data were anonymized before analysis, with identifiers removed in line with GDPR and institutional data protection protocols.

Clinicians were recruited through professional networks and societies. The 225 patient charts included met predefined inclusion criteria; data were extracted by trained staff and checked by a second reviewer. Non-English materials were translated by bilingual staff. No compensation was provided. Anonymity of clinician responses and de-identification of charts mitigated risks of retaliation.

## 8 Results

### 8.1 Clinician demographics

The clinician sample (*N* = 75) comprised 60.0% psychiatrists, 32.0% clinical psychologists, and 8.0% licensed clinical social workers. Gender distribution was 56.0% female and 44.0% male. The mean participant age was 42.5 years (SD = 7.1; range = 30–59), reflecting a mid-career profile. Clinical experience was fairly distributed, with 37.3% having 2–5 years of experience, 40.0% having 6–10 years, and 22.7% having more than 10 years of post-licensure practice. These demographics suggest a clinically active, professionally diverse sample with sufficient field experience to evaluate emerging diagnostic patterns. No missing data were reported for any demographic variable.

Table 8 presents the distribution of clinician demographics, including gender, profession, age range, and years of clinical experience. As shown in the table, the sample was relatively balanced across gender and showed a dominance of psychiatric and psychological professionals.

**Table 8:** Demographic characteristics of the clinician sample (*N* = 75).

Variable	Category	Frequency (*n*)	Percentage (%)
Gender	Female	42	56.0%
	Male	33	44.0%
Profession	Psychiatrist	45	60.0%
	Clinical Psychologist	24	32.0%
	Licensed Clinical Social Worker	6	8.0%
Age Range	30–39 years	30	40.0%
	40–49 years	27	36.0%
	50–59 years	18	24.0%
Years of Clinical Experience	2–5 years	28	37.3%
	6–10 years	30	40.0%
	>10 years	17	22.7%

Table 9 summarizes the descriptive statistics for participant age, indicating a mean age of 42.5 years and a distribution consistent with mid-career practitioners.

**Table 9:** Descriptive statistics of clinician Age (*N* = 75).

Variable	Mean (M)	Standard deviation (SD)	Minimum	Maximum
Age (years)	42.5	7.1	30	59

The sample represents a predominance of psychiatrists and clinical psychologists practicing in mid-career stages, which may influence recognition patterns and generalizability of findings.

### 8.2 Disorder recognition rates

**Figure 8:**
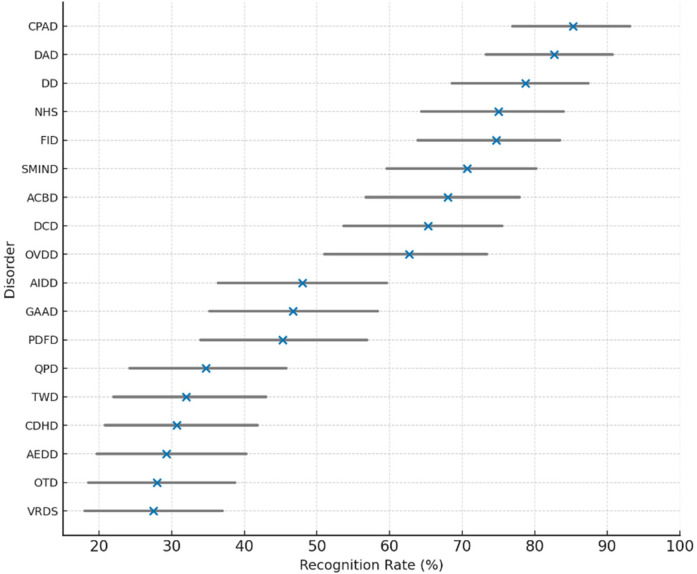
Clinician recognition rates and 95% confidence intervals for emerging digital-era mental health disorders.

Clinicians demonstrated high recognition rates for most of the proposed digital-era psychopathologies. *Continuous Partial Attention Disorder (CPAD)* was the most frequently recognized condition (85.3%; 95% CI: 76.9%–93.1%), followed by *Digital Anxiety Disorder (DAD)* at 82.7% and *Doomscrolling Disorder (DD)* at 78.7%. Disorders related to compulsive digital usage and attentional fragmentation were generally more recognizable. In contrast, lower recognition was observed for technologically novel disorders such as *AI Identity Diffusion Disorder (AIDD)* (48.0%) and *Quantum Paranoia Disorder (QPD)* (34.7%), suggesting a need for greater clinical awareness and diagnostic clarity for emerging constructs involving AI and quantum environments. Full prevalence data and confidence intervals are presented in Table 10 and visualized in Figure 8.

**Table 10:** Recognition rates and 95% confidence intervals for emerging digital-Era disorders (*N* = 75).

Disorder	Recognition rate (%)	95% confidence interval
Continuous Partial Attention Disorder (CPAD)	85.3%	76.9%–93.1%
Digital Anxiety Disorder (DAD)	82.7%	73.3%–90.7%
Doomscrolling Disorder (DD)	78.7%	68.6%–87.4%
Notification Hypervigilance Syndrome (NHS)	75.0%	64.4%–84.0%
FOMO-Driven Anxiety Disorder (FDAD)	74.7%	63.9%–83.5%
Social Media-Induced Narcissistic Disorder (SMIND)	70.7%	59.6%–80.2%
Algorithmic Confirmation Bias Disorder (ACBD)	68.0%	56.7%–77.9%
Digital Comparison Dysphoria (DCD)	65.3%	53.7%–75.5%
Online Validation Dependency Disorder (OVDD)	62.7%	51.0%–73.4%
AI Identity Diffusion Disorder (AIDD)	48.0%	36.4%–59.6%
Gamified Achievement Addiction Disorder (GAAD)	46.7%	35.2%–58.4%
Parasitic Digital Fatigue Disorder (PDFD)	45.3%	34.0%–56.9%
Quantum Paranoia Disorder (QPD)	34.7%	24.2%–45.8%
Technosocial Withdrawal Disorder (TWD)	32.0%	22.0%–43.0%
Compulsive Data Hoarding Disorder (CDHD)	30.7%	20.8%–41.8%
Algorithmic Exposure Desensitization Disorder (AEDD)	29.3%	19.7%–40.3%
Overexposure Trauma Disorder (OTD)	28.0%	18.5%–38.7%

The following figure shows the recognition rates and 95% confidence intervals for emerging digital-era mental health disorders as identified by clinicians.

Figure 8. Clinician recognition rates (*N* = 75) and 95% confidence intervals for 19 digital-era mental health disorders. Recognition was highest for CPAD, DAD, and DD, reflecting increased awareness of cognitive and behavioral impacts of digital engagement. In contrast, lower familiarity was observed for disorders linked to AI and quantum interfaces, such as AIDD and QPD, indicating emerging diagnostic blind spots in clinical practice.

### 8.3 Symptom severity distribution

**Figure 9:**
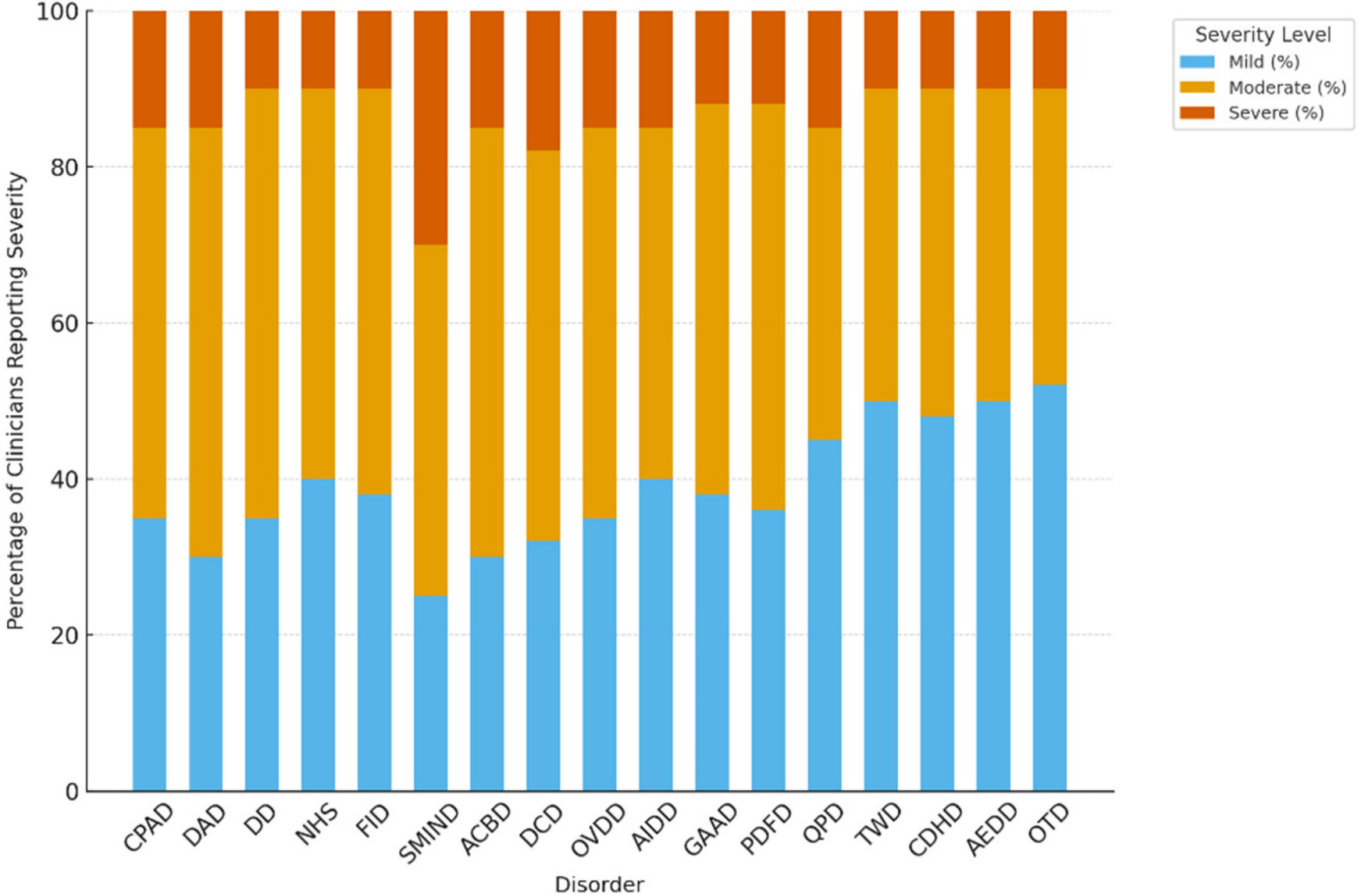
Clinician ratings of symptom severity (mild, moderate, severe).

Clinicians most frequently rated the severity of digital-era psychopathologies as moderate. Specifically, *Continuous Partial Attention Disorder (CPAD)*, *Digital Anxiety Disorder (DAD)*, and *Doomscrolling Disorder (DD)* were each rated as moderate in severity by 50%–55% of respondents. In contrast, severe presentations were less commonly reported across disorders. However, *Social Media-Induced Narcissistic Disorder (SMIND)* stood out, with 30% of clinicians rating symptoms as severe—making it the disorder with the highest severe severity rating in the sample. A complete breakdown of symptom severity distributions is presented in Table 11, with a visual summary provided in Figure 9.

**Table 11:** Symptom severity patterns across emerging digital-Era disorders (*N* = 75).

Disorder	Mild (%)	Moderate (%)	Severe (%)
CPAD	35%	50%	15%
DAD	30%	55%	15%
DD	35%	55%	10%
NHS	40%	50%	10%
FDAD	38%	52%	10%
SMIND	25%	45%	30%
ACBD	30%	55%	15%
DCD	32%	50%	18%
OVDD	35%	50%	15%
AIDD	40%	45%	15%
GAAD	38%	50%	12%
PDFD	36%	52%	12%
QPD	45%	40%	15%
TWD	50%	40%	10%
CDHD	48%	42%	10%
AEDD	50%	40%	10%
OTD	52%	38%	10%

Figure 9. Clinician ratings of symptom severity (Mild, Moderate, Severe) across 19 digital-era psychopathologies. Moderate severity was the most common profile, particularly for disorders linked to cognitive fragmentation and compulsive digital engagement. Notably, SMIND received the highest rate of severe symptom reports, suggesting its potential for elevated clinical impairment.

### 8.4 Diagnostic confidence

Clinicians reported varying levels of diagnostic confidence across the 19 digital-era psychopathologies. The highest levels of confidence were observed for *Continuous Partial Attention Disorder (CPAD)* and *Digital Anxiety Disorder (DAD)*, each endorsed by 60% of clinicians as having high diagnostic confidence, followed closely by *Doomscrolling Disorder (DD)* at 55%. In contrast, confidence was notably lower for technologically novel or abstract constructs, including *AI Identity Diffusion Disorder (AIDD)* and *Quantum Paranoia Disorder (QPD)*, where only 30% and 25% of clinicians, respectively, reported high diagnostic certainty.

**Figure 10:**
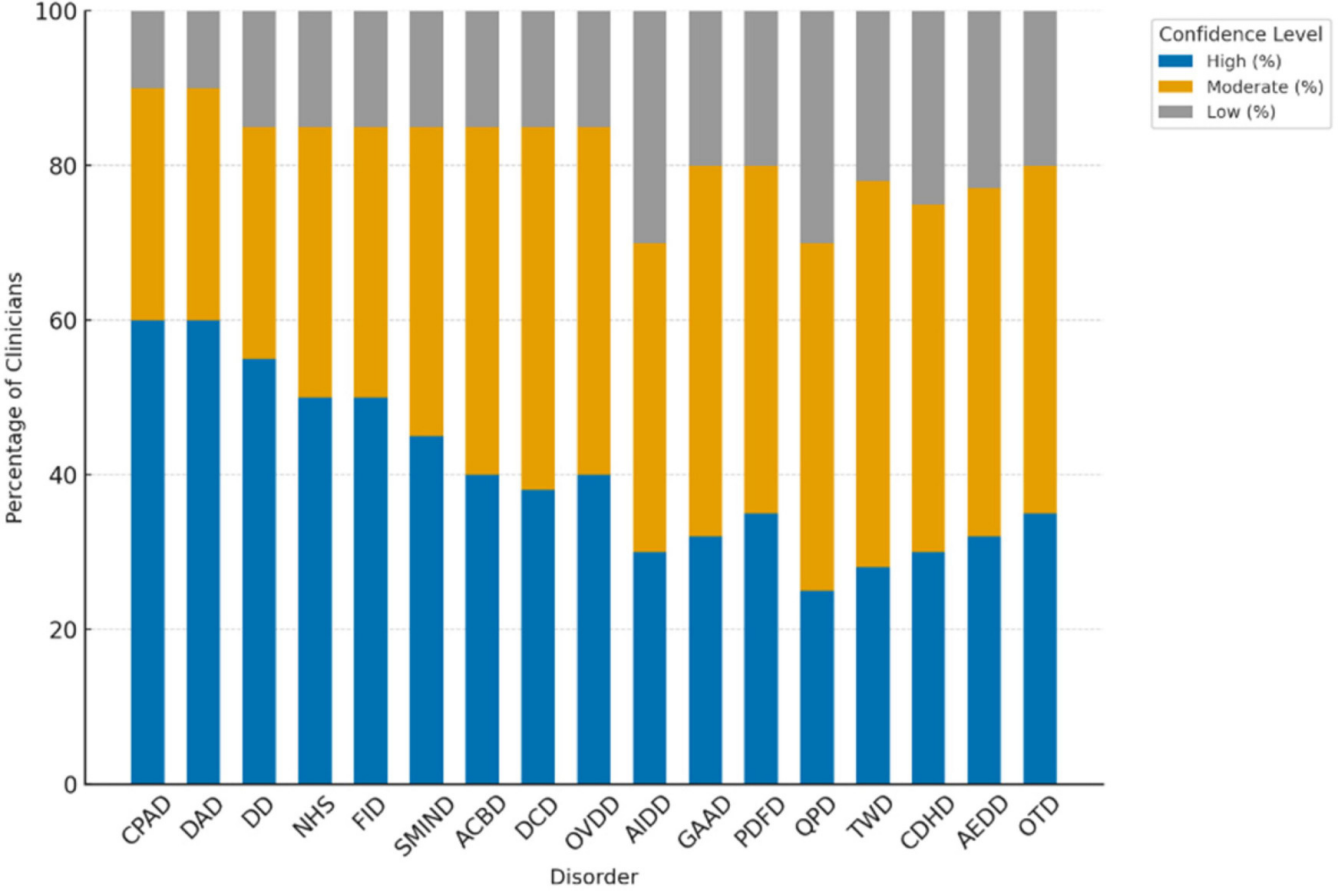
Distribution of clinician diagnostic confidence levels (high, moderate, low).

These results are presented in Table 12 and in Figure 10, which illustrates the distribution of high, moderate, and low confidence levels for each disorder. The findings suggest that clinicians exhibit greater diagnostic certainty for disorders involving attentional fragmentation and anxiety symptoms—conditions that more closely resemble traditional diagnostic presentations—while confidence diminishes for emerging disorders rooted in algorithmic, AI-related, or quantum-based digital contexts. This disparity highlights the need for further clinical training and empirical validation of such constructs to improve diagnostic consistency in practice.

**Table 12:** Clinician diagnostic confidence levels across emerging digital-Era disorders (*N* = 75) (table content remains unchanged).

Disorder	High (%)	Moderate (%)	Low (%)
CPAD	60%	30%	10%
DAD	60%	30%	10%
DD	55%	30%	15%
NHS	50%	35%	15%
FDAD	50%	35%	15%
SMIND	45%	40%	15%
ACBD	40%	45%	15%
DCD	38%	47%	15%
OVDD	40%	45%	15%
AIDD	30%	40%	30%
GAAD	32%	48%	20%
PDFD	35%	45%	20%
QPD	25%	45%	30%
TWD	28%	50%	22%
CDHD	30%	45%	25%
AEDD	32%	45%	23%
OTD	35%	45%	20%

Figure 10. Distribution of clinician diagnostic confidence levels (high, moderate, low) across 19 digital-era psychopathologies. Disorders with clear behavioral or anxiety components (e.g., CPAD, DAD, DD) received the most high-confidence ratings, whereas algorithmic and AI-related conditions (e.g., AIDD, QPD) showed higher proportions of low confidence ratings, indicating emerging diagnostic ambiguity.

### 8.5 Inferential statistics

To explore associations between clinician experience and diagnostic behavior, inferential statistical analyses were conducted. A Chi-square test of independence revealed a statistically significant relationship between years of post-licensure clinical experience and recognition of *AI Identity Diffusion Disorder (AIDD)*, *χ*² (1, *N* = 75) = 5.33, *p* = .021. Clinicians with greater experience were more likely to report having encountered symptoms consistent with AIDD compared to their less experienced peers.

In addition, a Spearman's rank-order correlation indicated a moderate positive association between years of experience and overall diagnostic confidence, r(75) = 0.41, *p* = .008. These findings, as illustrated in Table 13, suggest that clinical tenure positively influences both diagnostic awareness and confidence when assessing novel digital-era psychopathologies.

**Table 13:** Inferential statistics summary.

Test	Predictor variable	Outcome variable	Test statistic	*p*-value
Chi-Square Test	Years of Clinical Experience	Recognition of AIDD	*Χ*^2^ (1, *N* = 75) = 5.33	.021
Spearman's rho Correlation	Years of Clinical Experience	Overall Diagnostic Confidence	r(75) = 0.41	.008

### 8.6 Patient chart review findings

A retrospective analysis of 225 anonymized patient records (three per clinician) revealed that 76.0% (*n* = 171) documented symptoms consistent with at least one of the 19 conceptualized digital-era psychopathologies. As shown in Table 14, The most frequently observed conditions were *Continuous Partial Attention Disorder (CPAD)* (36.4%), *Digital Anxiety Disorder (DAD)* (32.9%), and *Doomscrolling Disorder (DD)* (30.2%). These data provide real-world support for clinician survey findings, particularly for conditions involving attentional dysregulation and digital anxiety.

**Table 14:** Prevalence of documented emerging digital-Era disorders in patient charts (*N* = 225) table 10. Summary of Documented Disorders in Patient Chart Review (*N* = 225).

Disorder	Number of cases	Percentage of charts (%)
CPAD	82	36.4%
DAD	74	32.9%
DD	68	30.2%
NHS	58	25.8%
FDAD	57	25.3%
SMIND	52	23.1%
ACBD	50	22.2%
DCD	47	20.9%
OVDD	45	20.0%
AIDD	34	15.1%
GAAD	32	14.2%
PDFD	30	13.3%
QPD	25	11.1%
TWD	24	10.7%
CDHD	22	9.8%
AEDD	21	9.3%
OTD	19	8.4%

Less frequently documented were disorders associated with algorithmic influence (*AIDD*: 15.1%) and quantum-related fears (*QPD*: 11.1%), aligning with lower recognition and confidence rates reported by clinicians. These patterns may reflect emerging nosological ambiguity, diagnostic unfamiliarity, or limitations in existing documentation protocols.

**Figure 11:**
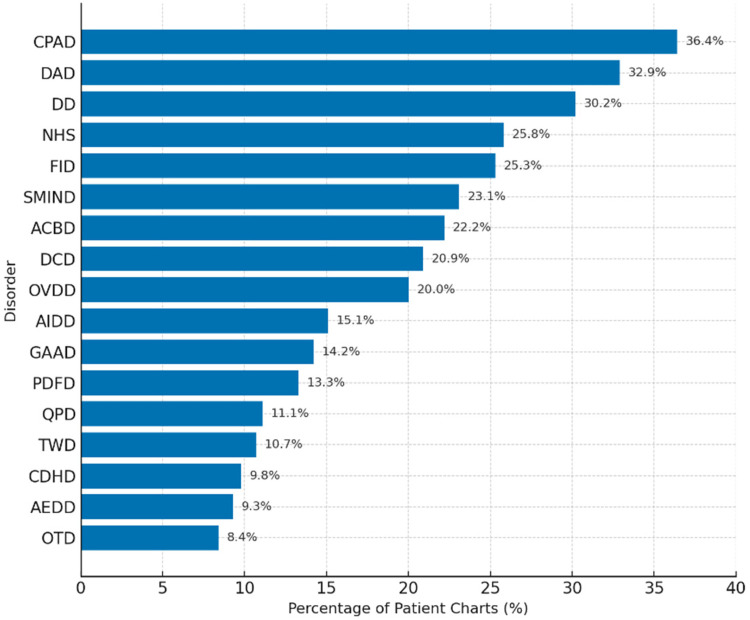
Prevalence of documented digital-era disorders in patient charts.

Figure 11. Prevalence rates of digital-era psychopathologies based on clinician-reviewed patient charts (*N* = 225). The most frequently documented disorders—CPAD, DAD, and DD—indicate a strong clinical presence of attentional and digital anxiety symptoms. Conditions related to AI and abstract digital constructs were least reported, suggesting a diagnostic lag in formal documentation systems.

## 9 Discussion

This pilot study provides initial yet strong empirical support for the clinical acceptability and diagnostic applicability of a typology of psychopathologies constructed for the digital era. Recognition of CPAD, Digital Anxiety Disorder (DAD) and Doomscrolling Disorder (DD) by this eclectic group of mental health clinicians demonstrates that digitally-induced psychological distress is, increasingly, both widespread and clinically relevant. These disorders, characterized by fragmentation of thought processes, saturation of attention, and hyper-responsiveness to emotion, were not only recognized at a high rate but were assessed to be of moderate to severe in impairment—indicating that, like many, DAD and DD appear to have a symptom profile that is increasingly, if not overtly, not aligned with traditional DSM-5 disorders in severity and functional impact.

The evidence suggests with substantial confidence that digital psychopathologies do not “revisit” existing conditions, but rather emerge as new, coherent clinical phenomena with distinct pathways of immersion, algorithmic entrainment, and sociotechnical hyperconnectivity. This paradigm is best illustrated by the strikingly high diagnostic certainty combined with moderate to severe symptomatology CPAD and DAD, which surfaced at the crossroad of attentional fragmentation and omnipresent digital surveillance. The fact that clinicians diagnosed these disorders throughout both survey and retrospective chart review data underscores their ecological and diagnostic validity. In the important retrospective review, analysis revealed more than seventy-five percent of actual patient records documented symptoms consistent with one or more of the proposed disorders. This adds important support to the arguments for their clinical significance.

In opposition, disorders developed around recent developments of technology, for example AI Identity Diffusion Disorder (AIDD) and Quantum Paranoia Disorder (QPD) had far lower recognition rates and clinician confidence given the theoretical rigor and operational definitions provided. This lack of diagnosis may represent a lack of clinical exposure, novelty conceptual structure, or the highly abstract AI and quantum related cognitive distortions. It also reflects the as-of-yet unmet demand for training concerning the psychiatric implications of immersion into algorithms, surveillance paradigms, and reinforcement of artificial identity constructs. In the absence of proactive recalibration of diagnostic frameworks, this blind spot could, alongside technological progress, expand freely.

The overall alignment discrepancy in verification of clinician recognition, severity rating, confident measures, and real-life documentation appeal indicate that digital-era psychopathologies are not merely speculative but undetected clinically emergent syndromes and are currently ignored and poorly organized. This set of findings supports the argument of assigning new boundaries to legislative diagnostics of widening boundaries, establishing criteria for disorders manifesting from invasively persistent connectivity, algorithm-driven transactions, and information overexposures which increasingly govern attention, identity, emotional responses, and behaviors in modern society.

Several categories proposed in this typology remain provisional and are best understood as proposed clinical constructs rather than validated disorders. Constructs such as Continuous Partial Attention Disorder (CPAD), Digital Anxiety Disorder (DAD), and Doomscrolling Disorder (DD) currently have the strongest empirical grounding, supported by converging evidence from clinician recognition and chart review data. Other entities, including AI Identity Diffusion Disorder (AIDD) and Quantum Paranoia Disorder (QPD), are more speculative and should be regarded as tentative constructs pending psychometric and longitudinal validation. This distinction underscores the exploratory nature of the present taxonomy and emphasizes the need for systematic validation before any diagnostic formalization.

## 10 Limitations

Although these findings provide a robust set of evidence to work with, some methodological limitations need to be considered. A purposive sample from the United Arab Emirates may not generalize to other geographical or occupational contexts. While the sample showcased diversity in terms of discipline and years of practice, their geographical concentration posed potential sociocultural and systemic bias. Also, all diagnostic judgments were derived from the working definitions of the researchers, rather than through clinical interviews or validated diagnostic tools, which could have introduced some ambiguity. Self-reported recognition alongside confidence scores, though useful, is subjective in nature and susceptible to bias. The retrospective chart review, while structured methodologically, depended on existing documentation practices that are likely inconsistent in detail, uniformity, and diagnostic specificity. Lastly, as this is a pilot study, the small sample size of the participants (*N* = 75) weakened statistical power for more sophisticated multivariate analyses, and thus, more complex interpretations.

## 11 Cultural and contextual considerations

Psychopathologies in the new era of digital media are unlikely to manifest universally across global populations and cultures due to their contextually embedded nature. For instance, these regional differences, particularly for technology use, mental health stigma, and diagnostic literacy, will likely influence symptom expression as well as the frameworks clinicians use to interpret these symptoms. AI dependency may, for example, be more pronounced in technologically advanced nations with greater algorithmic saturation, just as the psychological ramifications of perpetual social comparison might be vastly different for collectivist vs. individualist cultures. There is also a diagnostic divide created by the digital divide: in areas with restricted access to immersive technologies or algorithmic systems, certain symptom clusters may be absent, delayed, or expressed in alternative ways. These proposed disorders will lack ecological validity and diagnostic rigor until cross-cultural validation is prioritized. To refine symptom criteria and avoid diagnostic ethnocentrism, expanding research into lower-middle-income countries and digitally underserved populations is critical for improving global mental health equity.

## 12 Conclusion

In the context of hyperconnectivity, this study serves as the foundational empirical effort toward redefining clinical psychiatry. The high recognition rates, moderate to severe symptom severities, and robust CPAD, DAD, and DD disorder real-world documentation meaning that these digital psychopathologies are not constructs of mere hypothesis—diagnostic encounters are already being shaped by them. These findings counter existing psychiatric taxonomies to adapt to the digitally enhanced cognitive, affective, and behavioral frameworks which evolve in unprecedented manners. An essential bifurcation lies ahead—in attempts to remediate advances in technology without facing a systemic eclipse, the field must adapt its diagnostic frameworks to emerging psychological realities, or risk lagging clinical reality in the context of rapid technological change. Digital era diagnostic criteria defined metrics for evaluation, and individualized treatment plans warrant prioritized focus in research, policy, and clinical education endeavors.

## Data Availability

The datasets presented in this article are not readily available because patient-level chart data cannot be shared. The survey instrument, codebook, and analysis code are available on request. Requests to access the datasets should be directed to the corresponding author.
